# On the dilemma of using single EV analysis for liquid biopsy: the challenge of low abundance of tumor EVs in blood

**DOI:** 10.7150/thno.115131

**Published:** 2025-07-24

**Authors:** Meruyert Imanbekova, Mohul Sharma, Sebastian Wachsmann-Hogiu

**Affiliations:** Department of Bioengineering, McGill University, Montreal, QC, H3A 0E9, Canada.

**Keywords:** extracellular vesicles, liquid biopsy, single EV analysis, low abundance of tumor EVs, non-invasive cancer diagnostics

## Abstract

Single extracellular vesicle (EV) analysis holds great promise for non-invasive cancer diagnostics, offering insights into tumor-specific biomarkers and enabling personalized treatment strategies. However, a significant challenge in the path towards clinical applications is the low abundance of tumor-derived EVs (*tEV*s) in biofluids, which reduces the sensitivity, specificity, and accuracy of detection. This review emphasizes the importance of analyzing a large number of single EVs to overcome this limitation. We estimate that less than 0.1% of total EVs could be from cancer cells in a mixed sample. Additionally, the development of more efficient *tEV*s isolation methods and targeted enrichment strategies, as well as high-throughput analysis techniques are crucial for improving diagnostic accuracy and advancing liquid biopsy applications in cancer care.

## 1. Introduction

Cancer remains one of the leading causes of morbidity and mortality worldwide, underscoring the critical need for early and accurate diagnostic methods. Current technologies for detecting cancer, particularly in its early stages, have made significant progress, but there are still challenges to overcome. Early-stage cancer detection is crucial for improving survival rates, as tumors are often more treatable when detected early. Imaging techniques are well-established for tumor detection, but their ability to detect early-stage cancers is limited by tumor size, location, and metabolic characteristics. They are often more useful for staging and monitoring rather than for initial detection in asymptomatic patients. Biomarker-based immunoassays show promise for early detection, particularly in high-risk individuals, but they are not universally applicable across all cancer types. Their current role is more about monitoring and screening rather than providing definitive early detection. Traditional biopsy techniques can be invasive and may not always provide a comprehensive view of tumor heterogeneity 1.

**Figure 1:**
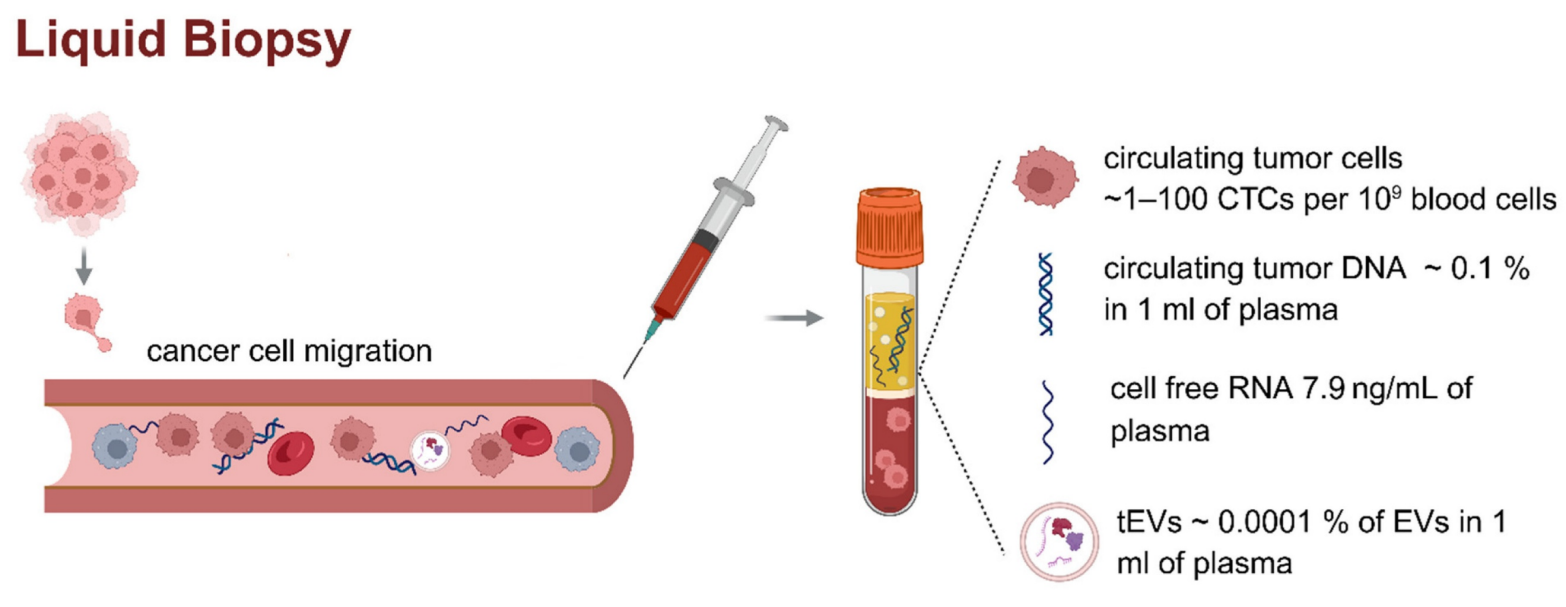
** Liquid biopsy and circulating biomarkers in plasma.** Liquid biopsy is a minimally invasive method for analyzing tumor-derived components such as ctDNA, cfDNA, cfRNA, and *tEVs* in peripheral blood. These biomarkers are typically present at very low concentrations (CTCs found in plasma ~ 1-100 CTCs per 10^9^ blood cells, ctDNA ~ 0.1 % in 1 ml of plasma, cfRNA concentration 7.9 ng/ml of plasma, concentration of *tEVs* ~ 0.0001 % of EVs in 1 ml of plasma), posing significant analytical challenges 4-6. The figure presents the literature values of the concentrations of individual circulating biomarkers in patient plasma samples. The low abundance of these analytes highlights the need for highly sensitive and specific technologies for clinical application in cancer diagnostics and monitoring. Created in https://BioRender.com.

Liquid biopsy, on the other hand, encompasses a range of minimally invasive techniques for analyzing tumor-derived materials in body fluids, including circulating tumor DNA (ctDNA), circulating tumor cells (CTCs), and cell-free RNA (cfRNA) (Figure 1) 2, 3.

Each of these biomarkers offers distinct advantages and limitations: ctDNA enables mutation profiling but is often present in low concentrations, especially in early-stage disease 5; CTCs provide rich phenotypic and genomic information, but are rare and difficult to isolate 7. In contrast, EVs are abundantly released by most cell types, are stable in circulation, and carry a diverse cargo of proteins, lipids, and nucleic acids that reflect their cell of origin 8. These properties make EVs an attractive and versatile source of biomarkers for disease diagnosis, prognosis, and monitoring, warranting further investigation in the context of liquid biopsy 9.

**Figure 2:**
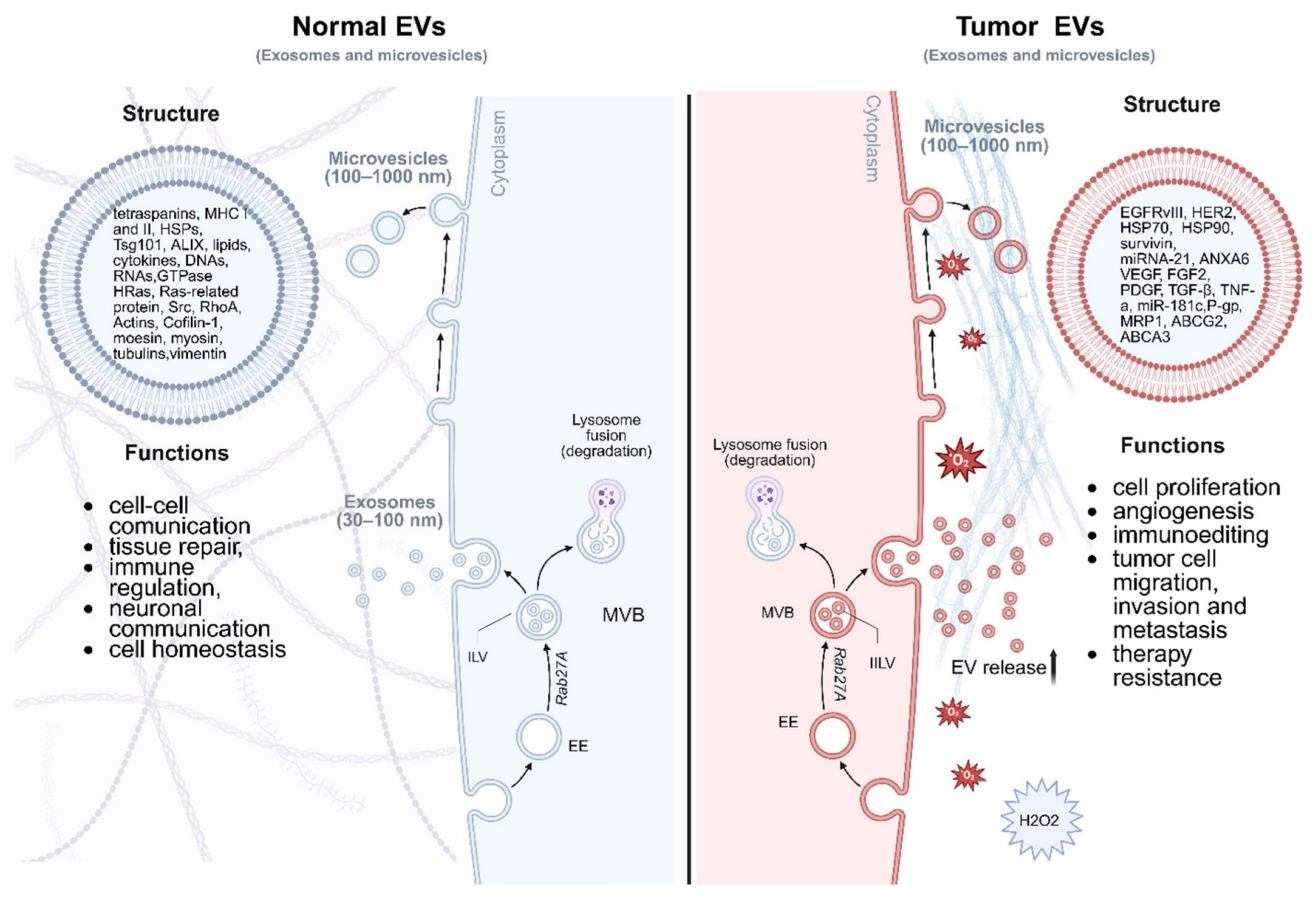
**Comparison of EVs derived from tumor cells and normal cells.** Both types of EVs share a common bilayer membrane structure and carry proteins, lipids, and nucleic acids; however, their content and functions differ significantly. Tumor EVs are typically enriched in oncogenic proteins such as EGFR and HER2, tumor-promoting miRNAs including miR-21, immunosuppressive molecules (PD-L1), supporting cancer progression, angiogenesis, immune evasion, and metastasis. In contrast, EVs from healthy cells contain homeostatic markers (CD9, CD63, CD81, TSG101), functional mRNAs, and regulatory miRNAs, and they play roles in maintaining tissue equilibrium, intercellular communication, and immune surveillance. Tumor cells exhibit an increased rate of EV compared to normal cells, thereby amplifying their paracrine and systemic effects within the tumor microenvironment (TME). The TME is frequently characterized by hypoxic conditions, elevated levels of reactive oxygen species such as hydrogen peroxide, and metabolic stress, all of which influence the biogenesis, cargo composition, and functional impact of tumor-derived EVs. Importantly, tumor-derived EVs also interact dynamically with the extracellular matrix (ECM), contributing to its biochemical and biomechanical remodeling. The ECM often undergoes pathological stiffening due to aberrant deposition and cross-linking of matrix components such as collagen and fibronectin. Tumor EVs can exacerbate this process by delivering matrix-modifying enzymes and fibrogenic mediators, thereby facilitating ECM remodeling that supports tumor invasion, angiogenesis, and metastasis. Early endosome - EE, multivesicular body - MVB, intraluminal vesicles - ILV, major histocompatibility complex class I - MHCI, HSP - heat shock protein, Annexin A6+ - ANXA6, vascular endothelial growth factor - VEGF, platelet-derived growth factor - PDGF, fibroblast growth factor 2 - FGF2, tumor necrosis factor alpha - TNF-α, P-glycoprotein - P-gp, multidrug resistance protein 1 - MRP1, Tumor Susceptibility Gene 101 - TSG10, ALG-2-interacting protein X - ALIX. Created in https://BioRender.com.

Nearly all cell types release these nanoscale lipid bilayer-enclosed particles that carry a wealth of information, including proteins and lipids. EVs are categorized mainly into three types: exosomes, microvesicles, and apoptotic bodies 8. They vary in size and origin and serve as crucial mediators of cellular and tissue functions 10. EVs are involved in numerous biological processes, including immune response 11, cellular signaling 12, and tissue repair 13. Importantly, the cargo within EVs reflects the physiological state of their parent cells, making them valuable biomarkers for disease diagnosis. *tEVs* exhibit distinct biological behaviors compared to EVs released by normal cells (*nEVs*), reflecting the altered molecular and functional state of malignant cells (Figure 2) 14. One of the most prominent distinctions lies in the upregulated release of *tEVs*, which is frequently driven by oncogenic signaling pathways such as RAS, EGFR, and MYC, as well as environmental stressors including hypoxia, low pH, and nutrient deprivation within the tumor microenvironment 15, 16. These factors collectively enhance EV biogenesis through modulation of the endosomal sorting complex required for transport (ESCRT) machinery and other vesicle formation pathways, resulting in increased vesicle production and altered cargo composition. In terms of uptake, *tEVs* demonstrate preferential interactions with specific recipient cells, including various immune cell subsets. This selective uptake is mediated by surface molecules such as integrins, tetraspanins, and phosphatidylserine, which facilitate vesicle internalization and determine cellular tropism 17.

Notably, *tEVs* have been shown to exert significant immunomodulatory effects. They are capable of delivering immunosuppressive cargo, including programmed death-ligand 1 (PD-L1), transforming growth factor-β (TGF-β), interleukin-10 (IL-10), and tumor-associated microRNAs such as miR-21, miR-23a, to immune cells 18. These factors collectively contribute to T cell exhaustion, inhibition of antigen presentation, expansion of regulatory T cells, and polarization of macrophages toward an M2-like immunosuppressive phenotype. Through these mechanisms, *tEVs* effectively facilitate immune evasion and promote tumor progression 19. Furthermore, the capacity of *tEVs* to modulate immune responses may also influence their pharmacokinetics. While *nEVs* are generally subject to rapid clearance by the mononuclear phagocyte system, particularly in the liver and spleen, *tEVs* may exhibit prolonged circulation times due to their ability to inhibit immune recognition and phagocytic uptake 20, 21. This extended half-life enhances their potential utility as circulating biomarkers in liquid biopsy applications.

The molecular and functional divergence between *tEVs* and *nEVs* also provides opportunities for the development of selective enrichment and targeting strategies. Surface proteins uniquely or preferentially expressed on *tEVs*, such as epithelial cell adhesion molecule (EpCAM), human epidermal growth factor receptor 2 (HER2), or mutant forms of epidermal growth factor receptor (EGFR), may serve as effective markers for immunoaffinity-based isolation. In addition, differential glycosylation patterns, lipid compositions, and nucleic acid profiles may offer alternative approaches for *tEV*-specific separation, including lectin affinity chromatography, aptamer-based capture, or size- and density-based fractionation. Exploiting these differences could significantly improve the sensitivity and specificity of EV-based diagnostics and enable the design of novel therapeutic strategies, including the targeted delivery of drugs or immune modulators via engineered vesicles.

Currently, research is focused on characterization of the size, shape, relative number, and cargo of EVs and detection of specific tumor-associated markers 22-24. While EVs from cancer and normal cell lines have been thoroughly evaluated, translating cell culture measurements into liquid biopsies has been challenging. Approaches such as EV isolation and enrichment (separation based on size, density, or surface markers) are commonly performed to separate EVs of a certain provenance, with label-free characterization being now explored in many reports, as detailed in the review article by Imanbekova *et al* 25. More recently, single EV analysis has gained interest due to the possibility of revealing the heterogeneity of EVs, thus providing deeper insights into disease mechanisms and potential therapeutic targets 26. In one of the first such demonstrations, Z. Smith *et al* demonstrated that Raman spectroscopy of optically-trapped single exosomes can reveal heterogeneity within EV populations that is often masked in bulk measurements 27. Single EV analysis can also detect low-abundance biomarkers that may be overlooked in traditional assays.

Cancer is one of the most promising areas for applications of single EV analysis. By focusing on single EVs, researchers can capture detailed information about the molecular characteristics of tumors, providing insights into the cancer's presence, type, and potential treatment pathways. For instance, in cancer diagnostics, single EVs can carry tumor-specific proteins or genetic material that signify the presence of malignancy 28. EVs from cancer cells often carry tumor-specific mutations, oncogenic proteins, and microRNAs, which can be used for early detection and monitoring of disease progression. For example, studies have shown that single EV analysis can detect KRAS mutations in pancreatic cancer and HER2 protein levels in breast cancer with high sensitivity 29, 30. This capability allows for not only early diagnosis but also the potential for personalized treatment strategies based on the molecular profile of the EVs. Single EV analysis can uncover this heterogeneity by allowing characterization of the molecular cargo of individual EVs. This can reveal different tumor subpopulations and their specific markers, which is crucial for tailoring personalized treatment strategies. For example, analyzing EVs from a heterogeneous tumor may identify distinct subclones that respond differently to therapies. The dynamics of EV release can also provide insights into tumor progression and treatment efficacy. As cancer evolves, the cargo of EVs may change, reflecting alterations in the tumor's biological behavior 28. By performing serial analysis of single EVs over time, clinicians could monitor how the tumor responds to treatment or whether it is developing resistance, potentially allowing for timely adjustments to therapy. One of the most compelling advantages of using single EV analysis for cancer diagnostics is the ability to obtain samples non-invasively. EVs can be isolated from readily accessible body fluids such as blood, urine, or saliva 31. This not only reduces patient discomfort but also allows repeated over-time sampling, which is essential for effective disease monitoring.

Advancements in technologies such as nanopore sensing, microfluidics, and high-resolution imaging techniques have significantly improved the ability to capture and analyze individual EVs 32. These innovations increase the likelihood of detecting rare and disease-specific biomarkers that could play a pivotal role in early diagnosis and personalized treatment. Despite the promise of these technologies, the number of EVs available for analysis remains a critical factor in the validity and reproducibility of results. For instance, the success of a single EV analysis depends not only on the ability to isolate and analyze EVs but also on the availability of an adequate number of *tEV*s in the sample to ensure statistical significance and robust findings.

Although numerous studies have reported promising results using single EV analysis, a consistent challenge is the limited quantity of *tEV*s obtained from biofluids, which can hinder the accuracy of biomarker detection, especially when trying to detect low-abundance markers 26. This issue is compounded by the inherent heterogeneity of EVs, where variations in size, content, and origin can make it difficult to draw definitive conclusions without large, well-characterized sample sizes. Furthermore, data interpretation remains complex, with the need to differentiate between EVs originating from the tumor versus those derived from other sources, such as immune cells or healthy tissues. Another limiting factor is the limited understanding of tumor-specific markers. While some tumor-specific proteins, RNAs, or other markers have been identified, there are still many unknowns regarding which markers on EVs are specific enough to reliably indicate the presence or progression of a particular type of cancer. Some markers may be expressed in multiple cancer types or in normal cells under certain conditions, complicating their use as definitive indicators.

This review aims to highlight the importance of the number of *tEV*s in liquid biopsy samples when single EV tools are employed. We use "single EV" to refer to the methodology used for the analysis of individual EVs, while "single EVs" refers to multiple vesicles analyzed individually, as opposed to EVs analysed in bulk. In the beginning of the article, we estimate the number of total EVs in liquid biopsy samples and perform simulations on the number of *tEVs* vs total EVs found in blood based on the number of cells, secretion rates, elimination rates, and uptake rates. We then discuss the use of different EV-containing biofluids. Next, we address EV isolation challenges, with a focus on the limited availability of *tEV*s and the need for enrichment methodologies specific for *tEV*s. In addition, we assess EV characterization methodologies for improved sensitivity and robustness. The discussion will then focus on the number of EVs required for accurate cancer detection, the limitations of single EV analysis techniques. Finally, we address the role of artificial intelligence (AI)-assisted data analysis and classification models in advancing the analysis of single EV analysis for cancer diagnosis by enabling automated and precise categorization of EVs based on their characteristics.

## 2. How Many EVs are Enough?

The total number of *tEV*s in analyzed samples is rarely evaluated and considered in single EV research. In a solid tumor, there may be hundreds of millions to billions of cancer cells 33. Cancer cells tend to release EVs at significantly higher rates than normal cells 34, 35. By using reported data of the number of EVs in 1 ml of blood of human prostate cancer patients, 10^4^ are tumor-specific 36, and the average total number of EVs in ml of human blood is 10^10^ 33, we calculated that only 0.0001% of total EVs could be from cancer cells in a mixed sample (Figure 1). These estimations are supported by the findings of Auber et.al., indicating that only <1% of EVs are derived from solid organs, and the other 99% of EVs from plasma are derived from immune cells and non-immune cells, including platelets and erythrocytes 37.

To make the matter more complicated, 1 ml of blood contains up to 10^6^ lipoproteins that may be misidentified as EVs, and up to 10^9^ platelets that continue secreting EVs 37-39. This makes detection of single *tEVs* in the blood even more challenging. In addition, a low abundance of tumor-specific biomarkers in *tEV*s can further hinder results interpretation. For example, a study of glioblastoma shows that less than one copy of most RNA species is present in *tEV*s. The levels of mRNAs that are most commonly mutated in glioblastoma were not higher than one copy per 100,000 EVs approximately, which indicates the necessity to appropriately interpret the data of EV RNA cancer biomarker research that is based on a single EV analysis 40.

In addition, estimating the rate of EV production is challenging because of the dynamic process associated with the *de novo* production and uptake of external EVs by any given cell type 41. The estimation of *tEV* secretion rates is complicated by the heterogeneity of tumors that have been shown to vary in cellular composition, genetic mutations, and phenotypic characteristics. The number of cancer cells can vary widely depending on the type and stage of cancer, which is shown to be reflected in EV secretion rates 42, 43. For example, the level of EVs in colorectal cancer and prostate cancer patients was statistically higher than that in healthy controls or benign tumor groups, and the numbers of EVs in plasma were associated with the degree of tumor differentiation and overall survival 44. Breast cancer cells shed lower numbers of EVs (~60 to 65 per cell per hour) compared with tissue-matched, nontumorigenic cell line-derived EVs 45. Several strategies and labeling methods are used to study EV secretion rates and their uptake, including fluorescent labeling, reporter gene systems, biotinylation, and stable isotope labeling. 41, 46-49. While mechanisms of EV uptake and cargo delivery are incompletely characterized, these studies show that EV uptake is highly regulated by the surface composition of EVs, may occur at different rates, and depends on the type of recipient cells.

To compare the number of *tEV* with that of *nEV* we can use the following formula:


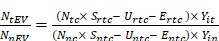
 (Eq 1)

where *N_tc_* and *N_nc_* are number of tumor cells and normal cells, respectively, *S_rtc_* and *S_rnc_* are the EV secretion rates from tumor and normal cells, respectively, *U_rtc_* and *U_ntc_* are the uptake rates for tumor and normal cells, respectively, *E_rtc_* and *E_ntc_* are elimination rates for tumor and normal cells, respectively, and *Y_it_* and *Y_in_* are the isolation yields of *tEV*s and *nEV*s, respectively.

**Figure 3:**
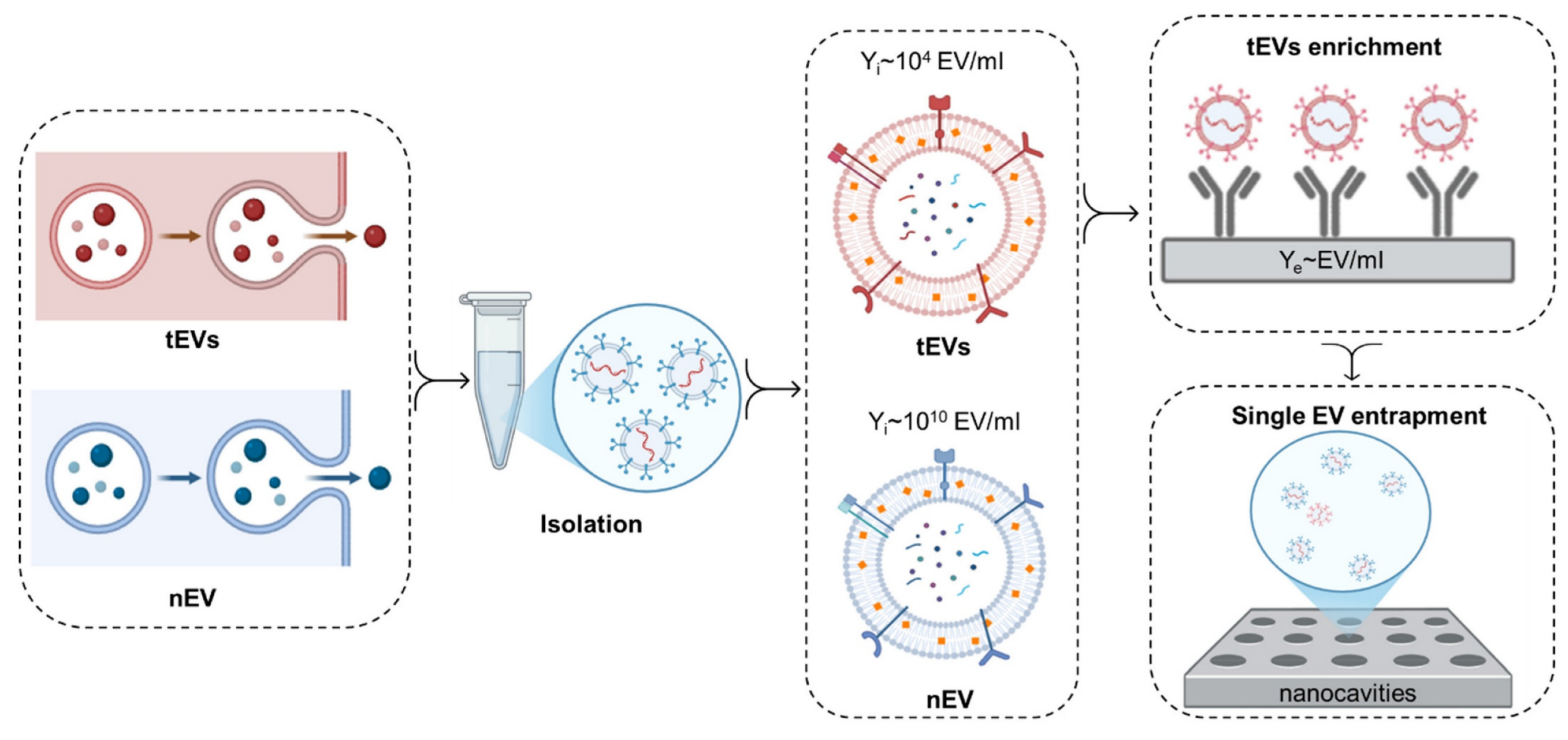
An overview of the critical factors affecting the efficacy of single EV analysis, such as EV release rates, isolation yields (*Y_i_*), and the importance of prior *tEV* enrichment. Cancer cell-derived EVs typically exhibit higher release rates, which can vary according to the tumor's type, grade, and microenvironmental factors. The release rates are quantified in terms of the number of EVs secreted per cell per day and are shown to correlate with tumor size and metabolic activity. Although total EV concentrations in 1 ml of body fluid can be in the range of 10^9^ to 10^10^ particles per milliliter, the proportion of *tEVs* is often low (<0.1 %). Therefore, it is crucial to implement enrichment strategies and single EV entrapment methodologies for increasing the proportion of *tEVs*, which, in turn, improves the detection of cancer-specific biomarkers.

This formula gives a more nuanced understanding of the abundance of *tEV*s versus *nEV*s by considering both the biological factors, including the number of tumor and normal cells, their secretion rates, and technical aspects such as the isolation efficiency. Notably, the secretion rates and isolation yields may significantly vary between tumor and normal cells. The number of *tEVs* for single EV analysis can be potentially enhanced by over 90% by selectively enriching *tEVs* 29, 50, 51. This has been demonstrated by S. Stott and collaborators, who achieved 94% *tEV* specificity in the detection of glioblastoma multiforme patients *via* immunocapture of EGFRvIII in plasma spiked with *tEVs* 50. Moreover, *Ferguson et al.* achieved 100% specificity in the detection of stage 1 pancreatic cancer, below the minimal tumor size for imaging-based detection, *via* single EV analysis through the detection of mutated KRAS and P53 proteins in endogenous EVs 29. Furthermore, *Min et al.* achieved, *via* single EV analysis of endogenous EVs, 100% specificity in detecting esophageal cancer *via* the CD36 marker, 95% specificity for stomach cancer *via* the TENM2 marker, 100% specificity for colorectal cancer *via* the CDH13 marker, 93% specificity for liver cancer *via* the TIMP2 marker, and 97% specificity for detecting lung cancer *via* the MUC1 marker 51. Selective enrichment can therefore lead to a more accurate reflection of the *tEV* population in the analysis, improving both the sensitivity and reliability of the results (Figure 3).

The rates of EV uptake and clearance are influenced by various factors, including the size, composition, and surface markers of the EVs, as well as the target cell type and the presence of specific receptors 52. EV uptake typically follows a dose-dependent pattern, meaning that larger quantities of EVs are more likely to be internalized, but the efficiency of uptake can vary based on the recipient cell's ability to recognize and internalize the vesicles. Immune cells, such as macrophages and dendritic cells, are particularly efficient at EV uptake due to their specialized role in maintaining immune homeostasis 53. The rate of clearance from the bloodstream, on the other hand, is largely determined by the reticuloendothelial system (RES), primarily the liver and spleen, which filter out circulating EVs. Larger or more negatively charged EVs are generally cleared faster due to greater recognition by phagocytic cells in these organs 54. In contrast, smaller EVs or those with camouflaged surface markers, such as those expressing certain proteins or lipid modifications, may evade rapid clearance, circulating for longer periods. Additionally, the half-life of EVs in circulation can vary depending on whether they are exposed to enzymatic degradation, endocytosis, or renal filtration. A study by *Matsumoto et al*. reported that the half-life of plasma-derived small EVs in circulation is about 7 min and was directly affected by the concentration of macrophages 53.

***Simulations of EV numbers in blood.*** Simulations were performed using Eq 1 to demonstrate the impact of varying different biological factors on the number and progression of blood plasma *tEVs* originating from breast cancer tumors. Contributions from various non-cancerous blood cell types were included in the calculations, including red blood cells, platelets, monocytes, and memory cells, for which secretion rates (in EVs/cell/min) and cellular quantities, i.e. *S_ntc_* and *N_nc,_* respectively, were acquired from Auber & Svenningsen 37. While organ-derived EVs were not considered during the simulations, their contribution to the total plasma EV makeup has been demonstrated to account for only ~1% of all plasma EVs. Additionally, *Bonsergent et al*. demonstrated the normal uptake of EVs to be a low-yield process, with a spontaneous rate of approximately 1%/hour, 0.01 hour^-1^, or ~0.0001667 min^-1^ 41. Pharmacokinetic analyses of blood plasma sEV (small EV) concentrations by *Matsumoto et al*. 53 also suggested an elimination half-life of 7 minutes, which can be converted into an elimination rate through pharmacokinetic considerations:


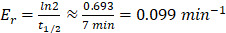
 (Eq 2)

For the purposes of the simulation, identical isolation yields of 1 were assumed between cancerous and non-cancerous EVs, thereby permitting *Y_it_* and *Y_in_* to be disregarded from the analysis. Consequently, for any given cell type, with the consideration of the secretion rate in EVs/cell/min and the uptake/elimination rates in the units of EVs/min, the rate of plasma EV inflow per minute (*R_inflow_*) can be found as follows:


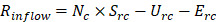
 (Eq 3)

However, with the aforementioned secretion and uptake rates being found in the units of min^-1^, a time-dependent analysis is evidently required. Consequently, *R_inflow_* at any given minute *k* can be found by computing the number of EVs secreted per minute, *N_c_* ✕* S_rc_*, and subtracting from that the product between the total number of EVs at the previous time point and the sum between the elimination and uptake rates:


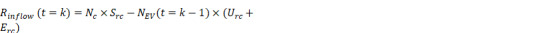
 (Eq 4)

Note that the first and second terms of Eq. 4 both give values in the units of EVs/min, thereby giving *R_inflow_* in EVs/min as well. As a result, the net number of plasma EVs at any given minute *k* can be found iteratively:


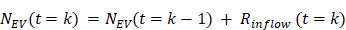
 (Eq 5)

or

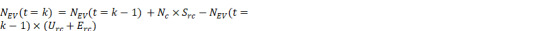
 (Eq 6)

Considering the aforementioned cellular quantities and secretion rates from Auber & Svenningsen 37, as well as the normal elimination and uptake rates of *E_R_* = 0.099 min^-1^ and *U_R_* = 0.0001667 min^-1^, the progressions in the number of plasma EVs originating from non-cancerous blood cell types were simulated using Eq. 6. Furthermore, the progression in plasma EVs originating from breast cancer cells was also simulated, considering the previously mentioned secretion rate of ~65 EVs/cell/hour 45. It is worth noting that this secretion rate approximation was acquired from breast cancer cells cultured in vitro. Consequently, this is likely an overestimation of the contribution to the total number of EVs by breast cancer cells in vivo, as in vivo secretion rates are likely to vary from those observed in vitro, and only a fraction of EVs secreted by tumors are transferred to plasma. Ultimately, our estimate constitutes an approximation for the purposes of demonstrating the effect of changing tumor size, tumor-EV uptake, and tumor-EV elimination. Notably, by estimating a tumor as a sphere with a density of 10^9^ cells/cm^3^, the diameter of the breast tumor could be directly related to the number of tumor cells, *N_tc_*. Furthermore, EV clearance from the blood has been demonstrated as being heavily dependent on phagocytic activity by immune cells 53. Considering that *tEVs* have been demonstrated as being capable of evading immune cells and impairing their function, it is likely that *tEVs* also exhibit slower blood plasma elimination rates 55. Consequently, elimination rates 0.1x and 0.01x the normal elimination rate were also tested to determine their effect on *tEV* progression. Finally, properties of the tumor microenvironment have been shown to promote *tEV* uptake, thereby suggesting that *tEVs* may exhibit higher than normal uptake rates, prompting an investigation into the effect of uptake rates 10x and 100x higher than normal on *tEV* progression 56.

**Figure 4:**
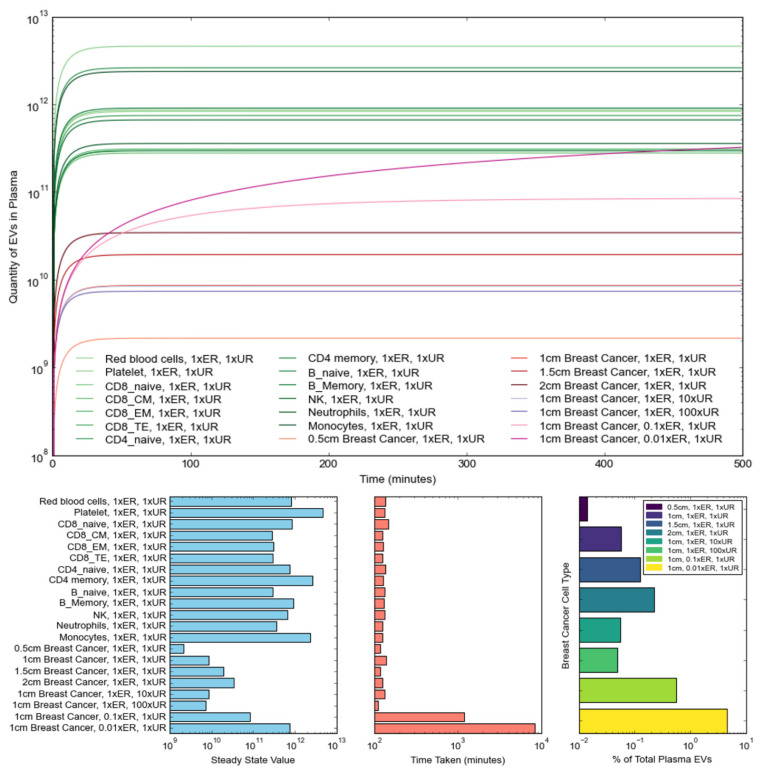
** Simulated progression of EVs in blood plasma originating from different cell types.** (Top) Variation in the quantity of EVs over 500 minutes, demonstrating the effect on EV progression of changing tumor size, uptake rate, and elimination rate for breast cancer cells; (Bottom-Left) Steady-state EV quantity reached for the different cell types; (Bottom-Middle) Time taken to reach steady-state EV quantities for different cell types; (Bottom-Right) Steady-state percentage contributions of breast cancer *tEVs* under different simulation conditions. Note that increasing tumor size shifts steady state concentrations of *tEVs* to higher values, without having discernible effects on the time taken to reach steady state. However, a ten-fold decrease of the elimination/clearance rate leads to a ten-fold increase in both the steady state concentrations of *tEVs* and the time taken to reach steady state. Uptake rates, on the other hand, have little effect on both the steady state* tEV* concentration and the time-to-steady-state.

The results obtained from the simulated investigations are shown in Figure 4, where the top graph shows the progression, in the blood plasma, of EVs originating from normal cells and breast cancer cells with varying tumor sizes, uptake rates, and elimination rates. The three bar graphs respectively showcase the steady-state EV quantities reached for each simulated cell type, the number of minutes taken to reach steady states, and the steady-state percentage contributions of breast cancer *tEVs* to the total makeup of plasma EVs under various simulation conditions. Notably, with regards to non-tumor cells, platelets, CD4 memory cells, and monocytes make up the primary contributors of net EV quantities in the plasma, consistent with the observations made by Auber & Svenningsen 37. Additionally, steady-state quantities were reached at relatively the same time for all normal cell types.

With regards to investigations performed in relation to breast-cancer-derived EVs, increasing tumor size had no significant impact on the speed at which steady state was reached, but instead shifted the curve upwards to higher steady state concentrations, likely as a result of the higher net secretion rates arising from the increased number of cells. Notably, increasing the tumor size from 0.5 cm in diameter to 2 cm in diameter resulted in an over ten-fold increase in the steady state number of tumor-derived EVs in the plasma. Opposingly, increasing uptake rate had no significant impact on both the steady-state number of *tEVs* and the speed at which the steady state was reached. Notably, there was a negligible decrease in the number of *tEVs* originating from a 1cm breast tumor when the uptake rate was increased from 1x*U_R_* to 100x*U_R_*, likely as a result of the basal uptake rate *U_R_* being relatively low to start with. Contrastingly, decreasing the elimination rate from 1x*E_R_* to 0.01x*E_R_* resulted in a significant increase in both the steady-state *tEV* quantity and the time taken to reach the steady state. As is showcased in Figure 4, each tenfold decrease in the elimination rate was accompanied by a tenfold increase in both the steady state *tEV* number and the time-to-steady-state, thereby suggesting that immune cell evasion and impairment is the primary means by which tumor EVs proliferate and promote tumor progression. Ultimately, the tumor size increase accompanied by tumor progression exacerbates *tEV* secretion further, with the increased *tEV* uptake rate having an almost strategically negligible impact on slowing *tEV* secretion in the blood plasma.

Furthermore, to evaluate the contribution of *tEVs* to the total plasma EV makeup, percentage makeups of *tEVs* under the tested simulation conditions were calculated as follows:


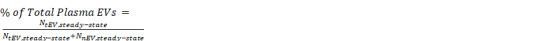
 (Eq 7)

In our case, *N_nEV, steady-state_* was estimated by adding up all the steady-state EV quantities of the simulated non-cancerous blood cell types. As previously mentioned, while these do not make up an exhaustive list of all sources of plasma EVs in the body, 99% of all plasma EVs are estimated to arise from these blood cells 37. That being said, as shown in Figure 4, the percentage contribution of *tEVs* does not typically rise above 0.1%, usually falling around the mark of 0.06%. It is noteworthy, however, that actual percentage contributions of *tEVs* are likely to be much lower with the addition of organ-derived EVs and with the consideration that only a certain percentage of *tEVs* is likely to leave the tumor microenvironment to enter the bloodstream. Notably, the uptake of *tEVs* in the blood and by surrounding cells, tumor cells, and immune cells is influenced by several factors, including the enhanced permeability and retention (EPR) effect 57. The EPR effect is a phenomenon that describes the irregular and leaky structure of tumor vasculature with large gaps between endothelial cells, leading to increased permeability 58. It allows macromolecules and nanoparticles to accumulate more easily within tumor tissue compared to normal tissues 59. Due to the leaky nature of tumor vasculature, EVs are more likely to accumulate in the tumor microenvironment, where they can be internalized by recipient cells through various mechanisms depending on the specific characteristics of the EVs and the type of target cell. This interaction can alter cellular behavior, promoting tumor cell proliferation, migration, and immune evasion. The EPR effect not only enhances the passive accumulation of EVs in the tumor but also improves their interaction with target cells in the tumor microenvironment, leading to a more effective cellular uptake 60.

Overall, the simulations presented in Figure 4 show that larger tumors result in a larger contribution of *tEVs* to the plasma, as generally expected. Additionally, it is once more notable that the strong impact of decreasing elimination rate on the proliferation of *tEVs*, with a 100x reduction in E_R,_ causing the percentage contribution to rise to almost 5%. While such low *tEV* elimination rates are highly unlikely, the impact of immune cell evasion and impairment is once more demonstrated as being an indispensable contributor to a tumor's progression and growth.

## ​3. Choosing the Optimal Biofluid for Single EV Analysis in Liquid Biopsy

The choice of the optimal biofluid for single EV analysis in liquid biopsy is critical to ensuring the sensitivity and accuracy of biomarker detection. Different biofluids, such as blood, urine, and saliva, contain varying concentrations and types of EVs, which can influence the detection of *tEV*s. The choice of biofluid must be carefully considered based on the stage of disease and the EV profile that best correlates with diagnostic and prognostic outcomes (Table 1).

While blood plasma in many cases is the biofluid of choice for *tEV* isolation due to its relatively higher EV yield and the ability to reflect systemic changes associated with cancer, urine and saliva are used to isolate EVs for prostate cancer detection and oral disease diagnosis, respectively. The anatomical proximity to the prostate makes urine a rational biofluid for prostate cancer diagnosis 66. This excretory biofluid is highly dynamic in terms of composition and depends on diet and medications, and requires pre-analytical steps to ensure consistent experimental results. Microbial contamination of urine may influence EV quantitation. In addition, the reported total number of EVs isolated from urine (10^9^ EVs/ml) is approximately the same as in blood 67.

Given its direct interaction with the oral environment, saliva is a biofluid of choice for oral cancer EV liquid biopsy. Reported concentrations of salivary EVs are slightly lower compared to blood, 10^8^ EVs/ml 68. As urine, salivary composition is highly dependent on medications and circadian rhythms 69.

## 4 Is the Enrichment of *tEVs* the Key to Unlocking the Potential of Single EV Analysis in Liquid Biopsy?

As our estimation shows, one significant challenge is the often-limited number of single *tEV*s that can be analyzed from a mixed sample of EVs in biofluids. Despite the high release rates of *tEV*s from cancer cells, the actual number of *tEV*s isolated and successfully analyzed may be low, particularly in early-stage diseases where the tumor burden is smaller. This limitation can result in insufficient data to make robust diagnostic conclusions or to capture the full spectrum of biomarkers present in EVs. Achieving high-purity *tEV* populations from complex biological fluids is critical for accurate analysis. Current isolation techniques, such as ultracentrifugation, polymer precipitation, and immunoaffinity capture, may lead to contamination with proteins and cellular debris, affecting the reliability of results. Inadequate purification can mask the true *tEV* population and hinder the detection of relevant biomarkers. Recently, new methods of isolation, including size exclusion chromatography, ultrafiltration, and immunocapture, showed higher purity in isolated EVs 70. In the single EV analysis of biofluids, prior enrichment of *tEV*s by employing immunocapture techniques targeting cancer-related or tissue-specific molecules can enhance specificity. For example, Melan A and MICA for melanoma 71, PSA for prostate cancer 66, L1CAM for brain tissue 72, epithelial cell adhesion molecule EpCAM, and HER2 are enrichment markers used for breast cancer 30. This approach of targeted enrichment of *tEVs* may yield over 90% *tEV* specificity across various cancer types, provided the correct biomarker is chosen 50, 73, 74. These promising results highlight the potential of selective enrichment techniques in improving the accuracy and sensitivity of single EV analysis of liquid biopsy. Additionally, selective enrichment through specific *tEV* biomarkers can assist in prognosis, on top of diagnosis, by targeting specific surface markers during immunocapture techniques. Examples of such markers include TMPRSS2 for bladder cancer, CPNE3 for colorectal cancer, and GPC1 for pancreatic cancer 75. However, the effectiveness of this approach can be challenged by the fact that the expression of tissue-specific and cancer-specific proteins in EVs is not fully understood. One of the major challenges in enriching *tEV*s for single EV analysis is the lack of a universal surface marker that can reliably distinguish these vesicles from others in the biofluid. While various surface markers mentioned above have been suggested for *tEV*s enrichment, none of these markers are universally expressed on all *tEV*s 76, 77. The challenges of this heterogeneity on selective enrichment are further exacerbated by the presence of active proteases on the surface of some EVs. Notably, these proteases can cleave surface proteins of EVs in a sample, potentially leading to the shedding of important surface biomarkers before they can be immunocaptured 78. Another potential type of tumor marker for *tEV* enrichment is neoantigens. Neoantigens are mutated proteins that arise from genetic alterations in tumor cells. These mutations, whether point mutations, insertions, deletions, or gene rearrangements, generate new peptide sequences that are absent in normal tissues. These altered peptides are displayed on major histocompatibility complex (MHC) molecules on the surface of cancer cells and can be recognized by the immune system as foreign. Neoantigens are particularly attractive for cancer immunotherapy because they are specific to the tumor and not present on normal cells. This makes them ideal targets for immune responses without causing widespread autoimmune effects. As tumors evolve, the landscape of neoantigens can change, which means that tumor-specific EVs could potentially carry different neoantigens depending on the tumor's stage, heterogeneity, and genetic evolution. Currently, EVs are mainly explored as immunotherapeutic agents for neoantigen loading and delivery 79. Yet, neoantigens in EVs can be utilized as potential biomarkers, increasing the specificity of EV-based diagnostics. In addition to neoantigens, there are several new promising marker types that are being actively studied for applications in EV analysis, such as glycosylation patterns, membrane lipid components, and non-coding RNAs. Notably, these new marker types display significant heterogeneity from *nEVs* to *tEVs* across different cancer cell types, offering diagnostic potential that has been extensively reviewed elsewhere 80-83.

Tumors exhibit heterogeneity in their EV populations, with different cancer types and even stages within the same type showing variable marker expression 84. Continued work on establishing universal and specific markers for enriching *tEV*s is essential to overcome a major hurdle that impacts the reproducibility and reliability of single EV analysis, particularly in liquid biopsy applications for early cancer detection. The lack of standardized markers for *tEV* enrichment is further complicated by the proteolytic cleavage of surface markers, which can lead to the loss or alteration of these markers on EVs, diminishing the effectiveness of enrichment strategies. This challenge underscores the need for more robust methods and markers that can consistently identify and isolate tumor-derived EVs, ensuring more accurate and reliable results in clinical applications 85.

Moreover, there is a pressing need for standardized protocols in EV isolation, characterization, and analysis. Variability in EV isolation methodologies can hinder reproducibility and the comparability of findings across different studies. Establishing consensus guidelines is essential to facilitate the adaptation of single EV analysis in clinical practice. The International Extracellular Vesicle Society addressed the initial lack of methodological consensus and reporting in the EV field by introducing several initiatives, such as MISEV guidelines and EV-TRACK platform.

The heterogeneity of EV populations complicates data interpretation. Single EV analysis can reveal diverse biomarker profiles, which may vary not only between different tumor types but also within the same tumor. A thorough understanding of the biological context of the analyzed EVs is essential for accurate diagnostic conclusions. Moreover, distinguishing between clinically relevant signals and background noise can be challenging, particularly with low-abundance biomarkers.

Beyond low-abundance and standardization issues, high-throughput single EV analysis faces other challenges to clinical translation. Notably, cost is variable depending on the methodology employed, where digital ELISA assays come at moderate costs compared to flow cytometry- and Raman spectroscopy-based high-throughput technologies, which incur high costs due to the need to purchase large, expensive equipment 86-88. However, such single EV analysis technologies are nevertheless expected to incur less cost than liquid biopsies focusing on ctDNA of CTCs 89. Liquid biopsies are typically slightly more expensive than traditional tissue biopsies, but often incur less cost compared to imaging techniques such as CT and MRI scans 90. Additionally, with regards to time required to perform diagnostic tests, liquid biopsy-based single EV analysis techniques are typically time-consuming, particularly due to the time required for isolating EVs, on top of the time needed for measurement and data analysis/interpretation 91. Ultimately, for single EV analysis to undergo widespread adoption as a non-invasive cancer diagnosis technique, further standardization is inevitably needed 91. Moreover, rather than competing with alternative liquid biopsy techniques involving ctDNA and/or CTCs, combined analysis of EVs, ctDNA, and CTCs offers significant synergistic potential, but has minimal clinical evidence and standardization, as well as further challenges arising from handling of the multimodal data 92.

## 5. Balancing Robustness and Sensitivity in Single EV Characterization Methods

To detect one copy of certain cancer biomarkers, including miRNA, a large number of single EVs must be analyzed 26, 93. This number varies depending on the biomarker of interest and the cancer stage. As shown previously, the levels of most commonly mutated mRNAs in glioblastoma (TP53 and PTEN) were approximately 1 molecule per 100,000 EVs, while most abundant mRNA species were present at 1 copy per 1,000 EVs 40. This threshold helps ensure that the heterogeneity of EV populations is adequately represented and that rare biomarkers are detected with sufficient confidence. However, for certain applications, such as monitoring specific mutations or protein expressions associated with particular cancers, significantly higher numbers may be necessary to improve statistical power and diagnostic accuracy.

Existing single-particle analysis techniques, including electron microscopy, surface plasmon resonance imaging (SPRi), super-resolution microscopy, fluorescent microscopy, and label-free plasmonic sensors, while being sensitive, are low-throughput, require expensive instrumentation, and are time-consuming (Table 2) 25. Therefore, the optimization of these methods is needed for the analysis of a large number of single EVs and to detect rare *tEV*s.

In addition, there are methods such as nanoparticle tracking analysis and flow cytometry that can analyze a large number of single EVs with high throughput. Flow cytometry is widely available in clinical laboratories, which makes it the most promising method for cancer EV liquid biopsy, as indicated in the recent review by Mizenko *et al* 111. Yet, the sensitivity of the technology remains to be improved to eliminate swarm detection. While traditional flow cytometers lack sensitivity to small nanoparticles and EVs, recent advancements of nano flow cytometers address this shortcoming and have been applied to analyse small nanoparticles, single EVs, and viruses. Liu H. and colleagues employed this emerging technology to perform in-depth characterization of DNA associated with single EVs. The study showed that localization of the DNA depends on the size of the EVs, revealing attachment of the DNA to the surface of the small EVs (<100 nm) and luminal localization of DNA in larger EVs (80 nm - 200 nm) 96.

In recent years, notable advancements were made in the efforts to tackle this fundamental challenge by developing innovative high-throughput single EV analysis platforms with the ability to analyze 20 million single vesicles per minute and limit of detection of 11 EVs/μl in blood 95. This technology is based on digital ELISA that utilizes paramagnetic, fluorescent beads coated with CD81 antibodies to capture single EVs. Another interesting approach for analysing a single EV is quantitative single-molecule localization spectroscopy (qSMLM) 112. This method had been applied to asses shape, size, tetraspanins content of single EV and explore their heterogeneity 109, 110. C. Han and colleagues developed single EV imaging methods based on total internal reflection microscopy to explore EV subpopulations 113.

Emerging high-throughput technologies for single EV analysis require robust data analysis capabilities to fully unlock their potential. The sheer volume of data generated by these platforms, combined with the inherent complexity of extracellular vesicle populations, necessitates the application of advanced computational tools to ensure accurate and meaningful interpretation. By implementing advanced machine learning algorithms, AI can analyze complex datasets generated from single EV characterization, such as size, shape, and molecular composition. This capability enables the identification of specific biomarkers associated with cancer, facilitating early detection and more accurate diagnoses. Additionally, AI-driven image analysis and pattern recognition techniques can significantly improve the sensitivity of detecting rare cancer-related EVs in biological samples, thus enabling personalized treatment strategies. AI classification models are pivotal in advancing the analysis of single EV analysis for cancer diagnosis by enabling automated and precise categorization of EVs based on their characteristics. These models often developed using machine learning techniques such as support vector machines (SVM), random forests, convolutional neural networks (CNN), transfer learning, and deep learning algorithms, can learn from large datasets to identify patterns that distinguish cancer-related EVs from normal ones. For example, SVM has been used to classify EVs based on their protein profiles obtained through mass spectrometry. By training on labeled datasets, SVM can effectively differentiate between EVs from cancer patients and healthy controls, highlighting specific biomarkers associated with tumor presence 114. CNNs have been applied to analyze imaging data from flow cytometry or nanoparticle tracking analysis. These models can automatically identify and classify EVs based on morphological features, enabling the detection of subtle differences that may indicate malignancy. CNNs have been used to classify EVs isolated from the 6 types of early-stage cancers based on Raman spectroscopy profile 115. Random forests have been utilized to analyze multi-omics data from EVs isolated serum of colon cancer, integrating information from RNA sequencing and proteomics 116, 117. Random Forests can classify EVs based on a combination of molecular signatures, providing a robust approach to identifying cancer-associated EVs. Next, advanced deep learning architectures, such as recurrent neural networks (RNNs) and autoencoders, have been explored for analyzing time-series data and high-dimensional feature sets from EVs 118. These models can detect dynamic changes in EV composition that correlate with tumor evolution, aiding in real-time monitoring of cancer.

As single EV data becomes more complex with the advancement of high-throughput technologies and the integration of multi-omics approaches, incorporating unsupervised clustering and dimensionality reduction pre-processing steps in model pipelines has become essential. It is widely accepted that biological data often suffers from the “curse of dimensionality”, wherein the number of data points required for a model to effectively form generalizations increases exponentially with the number of features 119. For biological datasets, such as transcriptomics and proteomics, the high dimensionality can significantly degrade model performance if proper pre-processing techniques like clustering and dimensionality reduction are not applied 119. This is also relevant to single EV analysis data, which often demonstrates high dimensionality that benefits from treatment with unsupervised learning algorithms 120-122. In essence, clustering methodologies identify instances with inherent similarities within the feature space, thereby partitioning the data to capture inherent subpopulations. Common clustering methodologies include K-Means Clustering, Hierarchical Clustering, and Expectation-Maximization Clustering. Notably, *Yin et al.* utilized K-Means clustering for their AI-assisted analysis of high-throughput SERS-based digital counting of single EVs for cancer diagnosis 120. Moreover, *Wen et al.* applied hierarchical clustering to their algorithm for the detection of plasma EV protein biomarkers for Ewing Sarcoma diagnosis via microfluidic Topographically-Intensified Partition-less dELISA 121. On the other hand, dimensionality reduction serves the purpose of transforming high-dimensional data into a low-dimensional representation that retains the most informative features, while discarding noise and redundancy. Some examples of dimensionality reduction techniques include linear methods such as Principal Component Analysis (PCA) and Independent Component Analysis (ICA), as well as non-linear techniques such as t-distributed Stochastic Neighbour Embedding (t-SNE) and Uniform Manifold Approximation and Projection (UMAP) 123. In addition to K-Means clustering, *Yin et al.* also utilized PCA for distinguishing between patients with different cancer types via SERS-based digital counting of single EVs 120. Moreover, *Von Lersner et al.* have applied UMAP in their machine-learning pipeline for analyzing and converting data from multiparametric single-vesicle flow cytometry into distinguishable EV fingerprints 122.

AI-assisted analysis techniques, when exploited in conjunction with high-throughput single EV detection methodologies, have also demonstrated significant potential in resolving heterogeneities within EVs. *Min et al.* trained a logistic regression model on single EV proteomic data of surface biomarkers from 100 cancer patients and 100 healthy patients, demonstrating the ability to distinguish between (i) healthy and cancerous patients, (ii) cancer type in cancerous patients (esophageal, stomach, colorectal, liver, or lung cancer), and (iii) intra-tumoral heterogeneity of EVs in colorectal cancer patients 51. Moreover, von *Lersner et al.* combined high-throughput multiparametric single vesicle flow cytometry with AI-assisted dimensionality reduction and clustering techniques to form EV fingerprints that help discern the partitioning of molecular cargo across different EV subpopulations 122. Furthermore, *Yin et al.* combined clustering and dimensionality reduction to create an algorithm that can potentially be used for distinguishing between patients with different cancer types via SERS-based digital counting of single EVs 120. However, the main obstacle in developing high-performing and versatile algorithms for resolving heterogeneity in single EVs remains a lack of annotated data, especially when considering the high-dimensionality of newly developed high-throughput techniques.

In addition, single EVs contain a wealth of information, including proteins, lipids, mRNA, and DNA. AI models can integrate these multiple types of data, generating more comprehensive insights. Multi-omics approaches such as proteomics, transcriptomics, and genomics allow for deeper understanding and cross-validation of cancer markers at different molecular levels, helping to pinpoint the most informative biomarkers for early detection. AI can also be used to merge data from different experimental platforms (flow cytometry, mass spectrometry, RNA sequencing). This data fusion enables researchers to better understand the complexity of cancer-derived EVs and identify comprehensive molecular signatures that may indicate the presence of early-stage cancer. Moreover, AI can be used to enhance the real-time detection of *tEVs* using microfluidic platforms and biosensors. Such technologies could potentially isolate individual EVs and analyze them in real time, while AI models process the data to classify EVs as cancerous or normal immediately. This real-time analysis is especially valuable for monitoring treatment responses or detecting recurrences.

While there are exciting avenues for AI application in single EV research, a lack of large, annotated datasets for training machine learning models can lead to data imbalance and reduce model performance, especially for rare cancers or early-stage diseases. Also, it is essential to develop ML models with deeper integration with domain-specific biological knowledge.

Overall, the integration of AI in single EV analysis not only streamlines diagnostic workflows but also holds the potential to revolutionize cancer management through more targeted and effective therapeutic interventions.

## 6. Conclusions

Single EV analysis represents a groundbreaking advancement in liquid biopsy technology, offering unprecedented opportunities for disease diagnosis. By enhancing sensitivity and specificity, it has the potential to transform the diagnostic landscape for various diseases, including cancer. This non-invasive method may offer detection of tumor-specific biomarkers with high sensitivity and specificity. The ability to analyze individual EVs provides valuable insights into the diverse characteristics of tumors, facilitating the development of personalized treatment strategies based on specific molecular profiles. However, a critical challenge in implementing single EV analysis is the availability of a sufficient number of *tEV*s for analysis. Despite the promise shown by EVs, our analysis indicates that current studies based on the analysis of hundreds of single EVs may lack robust statistical power and accurate representation of EV heterogeneity. For certain applications, particularly those monitoring specific mutations or protein expressions, even higher numbers of single EVs may be necessary to enhance diagnostic accuracy. Given the vast number of EVs released from a variety of cell types, including the immune cells, platelets, and erythrocytes, the concentration of cancer-specific EVs in a typical plasma sample is extremely low.

To address this challenge, in this article, we introduced a simulation model that helps provide an estimate of the abundance of *tEVs* among the total number of EVs. This model accounts for variabilities in the EV secretion, elimination, and uptake rates, as well as for the cell type and isolation yields. Using this simulation model, we estimate that no more than ~0.1% of the total EVs in a blood sample might originate from cancer cells, making it exceptionally difficult to capture and identify a single tumor-derived EV in a mixed sample. This low abundance is further complicated by the heterogeneity of tumors and the dynamic nature of EV production and uptake by different cell types. One important question that still needs to be addressed is whether studies analyzing hundreds or thousands of single EVs are inherently biased towards the detection of soluble tumor-associated markers within the analyte, or do they more accurately reflect alterations in the immune landscape of cancer patients rather than detecting cancer biomarkers associated with *tEV*s.

Considering these factors, it is critical in single EV studies to analyze a large number of single EVs in order to reliably detect tumor-specific EVs and associated cancer biomarkers. Therefore, for the identification of the extremely small fraction of cancer EVs, large-scale EV analyses are necessary, with the number of single EVs to be analyzed depending on the expected concentration of tumor-derived EVs. In practice, the number of EVs to be analyzed (with single EV techniques) should be at least 1 million per test to ensure that rare, low-abundance cancer-specific EVs are sufficiently represented. This is especially important for ensuring accurate biomarker analysis, given the low levels of tumor-specific RNA or proteins often found within these EVs. The number we provide here can be further refined by performing additional, detailed, and specific simulations in order to capture the prevalence of *tEVs* in different diagnostic circumstances, such as other tumor types, cancer stage, or biofluids. While improvements to the simulation model will be necessary to perform more accurate estimates, the main challenge is the availability of accurate data obtained from *in vivo* measurements. In this context, EV subtype-specific rates that are measured in relevant environments will be needed.

Furthermore, similar recommendations apply for other biofluids such as urine and saliva, although factors like microbial contamination and the highly variable composition of these fluids may further influence EV quantification. Despite these challenges, increasing the number of single EVs analyzed remains the most effective strategy for overcoming the low abundance and variability inherent in tumor-derived EV detection, paving the way for more accurate liquid biopsy diagnostics for cancer.

To propel the field forward, it is imperative to improve EV isolation and enrichment techniques to capture adequate amounts of *tEV*s from complex biological fluids, as well as develop high-throughput characterization techniques. However, post-processing of high-throughput single EV data necessitates the incorporation of machine learning pipelines for efficient and thorough consideration of inter- and intra-tumor heterogeneity within EV subpopulations. As research continues to refine these methodologies, single EV analysis could transform cancer diagnosis and monitoring, offering hope for early detection and improved patient outcomes. Efforts should also be placed to overcome accessibility barriers to clinical translation, including diagnostic time frames, cost, and standardization. Ultimately, while single EV analysis is not yet fully realized as a standalone or complementary diagnostic tool, it holds significant promise as part of a comprehensive approach to cancer care.

## Figures and Tables

**Table 1:** Comparison of optimal body fluids for single EV analysis in liquid biopsy

Biofluid	Advantages	Disadvantages	Applications	References
Plasma	high EV concentration rich in *tEVs* widely used clinically	high background of lipoproteins/proteins co-isolation of contaminants hemolysis may affect results	cancer diagnostics, biomarker discovery, monitoring treatment	61
Urine	non-invasive low protein background	low EV concentration diluted samples variable EV content depending on hydration	urological cancers	62
Saliva	non-invasive easy sampling reflects oral and systemic conditions	low EV concentration high bacterial content contamination with mucins, high viscosity challenges EV isolation	oral cancers	63
CSF	high relevance for CNS diseases	invasive collection low volume available	brain tumors	64
Pleural/Ascitic Fluids	abundance of *tEVs* relevant for metastatic cancers	invasive patient-specific variability	ovarian, lung, and gastrointestinal cancers	65

**Table 2:** Single EV characterization methods

Method	Advantages	Disadvantages	Detection Limit	References
flow cytometry	high-throughput multiparametric analysis surface marker-specific detection using fluorescent antibodies	limited sensitivity to small EVs (<100 nm), specificity depends on antibody quality and labeling signal overlap and background can affect accuracy	~100-150 nm	94-96
NTA	size distribution of individual EVs high-throughput fluorescent mode allows limited marker-specific detection	low resolution for heterogeneous or small EVs requires large sample volumes	~30-100 nm	97, 98
AFM	high spatial resolution label-free morphological analysis high specificity for physical properties (stiffness, size, surface structure)	low throughput technical expertise required	<10 nm	99
Raman spectroscopy/SERS	label-free chemical composition high biochemical specificity	low throughput sensitive to noise and sample preparation artifacts		27, 100, 101
TEM	high morphological resolution high specificity for EV structure and morphology selective molecular identification via immunogold labeling enables	labor-intensive low throughput	<10 nm	102
Super-Resolution Microscopy	allows investigation of EVs functions *in vivo/in situ* with molecular resolution	requires labels		103, 104
SPRi	real-time monitoring of EVs-ligand binding kinetics, small sample size, label free	labor intensive, limited by use of capturing molecules		105, 106
Digital ELISA	detection of EV surface or cargo proteins	requires optimized antibody pairs	<100 EVs/mL	95, 107
Nano-FTIR	label-free quantitative and qualitative characterization of EV biochemical content, minimal preprocessing, small sample size	technically demanding low throughput	~50-100 nm	108
qSMLM	Quantitative characterization of the size, shape and protein content	requires optimized antibody pairs, low throughput, complex sample preparation, and limited multiplexing capabilities	~10 nm	109, 110

## Abbreviations

EV: extracellular vesicle
*tEV*s: tumor-derived EVs
ctDNA: circulating tumor DNA
CTCs: circulating tumor cells
cfRNA: cell-free RNA
EGFR: epidermal growth factor receptor
ESCRT: endosomal sorting complex required for transport
HER2: human epidermal growth factor 2
TME: tumor microenvironment
ECM: extracellular matrix
MHCI: major histocompatibility complex class I
HSP: heat shock protein
ANXA6: annexin A6+
VEGF: vascular endothelial growth factor
PD-L: programmed death-ligand 1
TGF-β: transforming growth factor-β
IL-10: interleukin-10
EpCAM: epithelial cell adhesion molecule
RES: reticuloendothelial system
Melan A: melanoma antigen
MICA: MHC class I chain related-proteins A
PSA: prostate specific antigen
TMPRSS2: transmembrane protease serine 2
CPNE3: copine 3
GPC: glypican-1
SPRi: surface plasmon resonance imaging
qSMLM: quantitative single-molecule localization spectroscopy
SERS: surface-enhanced Raman spectroscopy
SVM: support vector machines
CNN: convolutional neural networks
RNNs: recurrent neural networks
ELISA: enzyme-linked immunosorbent assay
PCA: principal component analysis
ICA: independent component analysis
t-SNE: t-distributed stochastic neighbour embedding
UMAP: uniform manifold approximation and projection
